# Unraveling the Regulation of Hepatic Gluconeogenesis

**DOI:** 10.3389/fendo.2018.00802

**Published:** 2019-01-24

**Authors:** Xueping Zhang, Shanshan Yang, Jinglu Chen, Zhiguang Su

**Affiliations:** Molecular Medicine Research Center and National Clinical Research Center for Geriatrics, West China Hospital, State Key Laboratory of Biotherapy, Sichuan University, Chengdu, China

**Keywords:** gluconeogenesis, hormone, transcription factor, methylation, acetylation, AMPK, metformin

## Abstract

Hepatic gluconeogenesis, *de novo* glucose synthesis from available precursors, plays a crucial role in maintaining glucose homeostasis to meet energy demands during prolonged starvation in animals. The abnormally increased rate of hepatic gluconeogenesis contributes to hyperglycemia in diabetes. Gluconeogenesis is regulated on multiple levels, such as hormonal secretion, gene transcription, and posttranslational modification. We review here the molecular mechanisms underlying the transcriptional regulation of gluconeogenesis in response to nutritional and hormonal changes. The nutrient state determines the hormone release, which instigates the signaling cascades in the liver to modulate the activities of various transcriptional factors through various post-translational modifications like phosphorylation, methylation, and acetylation. AMP-activated protein kinase (AMPK) can mediate the activities of some transcription factors, however its role in the regulation of gluconeogenesis remains uncertain. Metformin, a primary hypoglycemic agent of type 2 diabetes, ameliorates hyperglycemia predominantly through suppression of hepatic gluconeogenesis. Several molecular mechanisms have been proposed to be metformin's mode of action.

## Introduction

Glucose functions as a primary fuel to active tissues like brain and blood cells in mammals and its blood levels are maintained within a relatively narrow range (1). The liver plays a crucial role in maintaining glucose homeostasis, as it is the main organ for glucose storage in the form of glycogen, as well as endogenous glucose production by glycogenolysis and gluconeogenesis. During short-term fasting periods, the liver produces and releases glucose mainly through glycogenolysis, which is the breakdown of glycogen to glucose. As the starvation time is prolonged, glycogen is depleted, and gluconeogenesis, that is *de novo* glucose synthesis from available precursors, becomes the main method of maintaining blood glucose levels. However, the abnormally increased rate of hepatic gluconeogenesis contributes to hyperglycemia of both type I and II diabetes. This underscores the importance of maintaining normal gluconeogenic rates to avoid disease pathophysiology. Therefore, identification of the molecular mechanisms regulating hepatic gluconeogenesis is crucial to the development of improved therapeutic strategies for the treatment of diabetes.

Gluconeogenesis is modulated by various external factors, such as nutrient and energy conditions, exercise, and stress reaction, through mediating the secretion and activity of certain molecules (2–4). The regulation of gluconeogenesis occurs on multiple levels, such as hormone secretion, gene transcription, and posttranslational modification. In response to the stimulation of external factors, hormone signals are promoted or inhibited, such as insulin, glucagon, and glucocorticoid, which in turn modulate the gluconeogenic pathways, regulating gene expression, and glucose production.

In this review, we would like to discuss the molecular mechanisms underlying the transcriptional regulation of gluconeogenesis in response to hormonal changes.

## Gluconeogenesis and its Unique Enzymes

**Figure 1:**
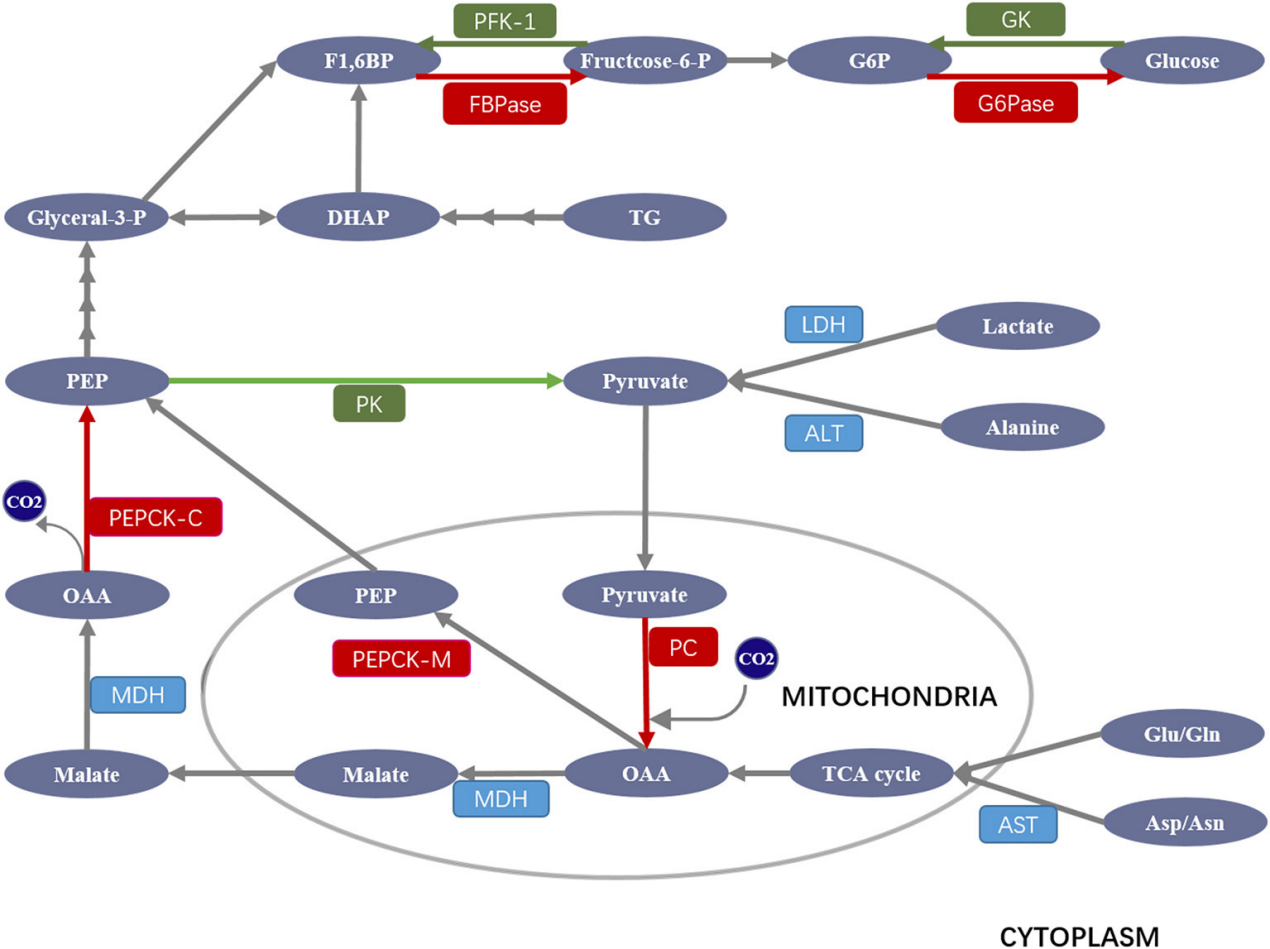
Schematic overview of major enzymes and metabolites involved in the regulation of gluconeogenesis. Pyruvate derived from lactate and alanine enters the mitochondrion, where it is carboxylated to oxaloacetate (OAA) by pyruvate carboxylase (PC). OAA is then reduced to malate to be shuttled to the cytoplasm where it is reoxidized to OAA, which is decarboxylated and then phosphorylated to phosphoenolpyruvate (PEP) by PEP carboxykinase (PEPCK). PEP then enters the gluconeogenic cycle. After several steps of reverse glycolysis, the yield fructose 1,6-bisphosphate (F1,6BP) is dephosphorylated by fructose 1,6-bisphosphatase (FBPase) to form fructose 6-phosphate, which is then converted to glucose-6-phosphate (G6P) by phosphoglucoisomerase. G6P is finally converted to glucose via dephosphorylation by glucose-6-phosphatase (G6Pase). Other gluconeogenic amino acids (Asp/Asn and Glu/Gln) are converted into alanine or specific intermediates in the tricarboxylic acid (TCA) cycle for gluconeogenesis. Glycerol is converted to dihydroxyacetone phosphate (DHAP), which is then converted to glyceraldehyde-3-phosphate (Glyceral-3-P) or F1,6BP entering the gluconeogenic pathway. ALT, alanine aminotransaminase; AST, aspartate aminotransaminase; GK, glucokinase; LDH, lactate dehydrogenase; MDH, malate dehydrogenase. Gluconeogenic and glycolytic enzymes are highlighted in red and green, respectively.

Gluconeogenesis is a process that transforms non-carbohydrate substrates (such as lactate, amino acids, and glycerol) into glucose (Figure 1). Both lactate and alanine are first converted into pyruvate, which then enters the mitochondrion and is carboxylated to oxaloacetate (OAA) by pyruvate carboxylase (PC). OAA is then reduced to malate to be shuttled to the cytoplasm where it is again reoxidized to OAA, which is decarboxylated and then phosphorylated to phosphoenolpyruvate (PEP) by cytosolic PEP carboxykinase (PEPCK-C). In addition to the cytosolic PEPCK, recent studies suggest that mitochondrial OAA can be directly converted to PEP by mitochondrial PEPCK (PEPCK-M) and then shuttled to the cytoplasm (5, 6). PEP then enters the gluconeogenic cycle. After several steps of reverse glycolysis, the yield fructose 1,6-bisphosphate (F1,6BP) is dephosphorylated by fructose 1,6-bisphosphatase (FBPase) to form fructose 6-phosphate, which is then converted to glucose-6-phosphate (G6P) by phosphoglucoisomerase. Finally, G6P is converted to glucose via dephosphorylation by glucose-6-phosphatase (G6Pase). Notably, other gluconeogenic amino acids, such as aspartate and glutamate, are converted by less direct routes into alanine or specific intermediates in the tricarboxylic acid (TCA) cycle for gluconeogenesis. Glycerol, released into plasma from adipose tissue, is taken up into the liver where it is converted to dihydroxyacetone phosphate (DHAP), which is then converted to glyceraldehyde-3-phosphate (Glyceral-3-P) or F1,6BP, entering the gluconeogenic pathway.

Although gluconeogenesis is theoretically the reversal of glycolysis, there are three key irreversible glycolysis kinase reactions catalyzed by glucokinase (GK), phosphofructokinase-1 (PFK-1), and pyruvate kinase (PK) (7), which are overcome by four unique gluconeogenic enzymes including PC, PEPCK, FBPase, and G6Pase (Figure 1). Given their importance in gluconeogenesis, the deregulation, or deficiency of these four enzymes can cause serious diseases, including type 2 diabetes in humans. For example, inhibition of hepatic PC not only decreases glycerol synthesis in adipose and liver, it also improves hepatic insulin signaling, while PC overexpression stimulates gluconeogenesis and leads to hyperglycemia (8); PEPCK deficiency contributes to a diverse level of persistent neonatal hypoglycemia and liver dysfunction (9); FBPase deficiency frequently causes hypoglycemia (10), while its overexpression leads to hyperglycemia and impaired insulin secretory function (11); G6Pase deficiency can lead to glycogen storage disease (GSD) with hypoglycemia, hepatomegaly, lactic acidemia, hyperuricemia, and hyperlipidemia (12).

## Hormones Control Gluconeogenesis

**Figure 2:**
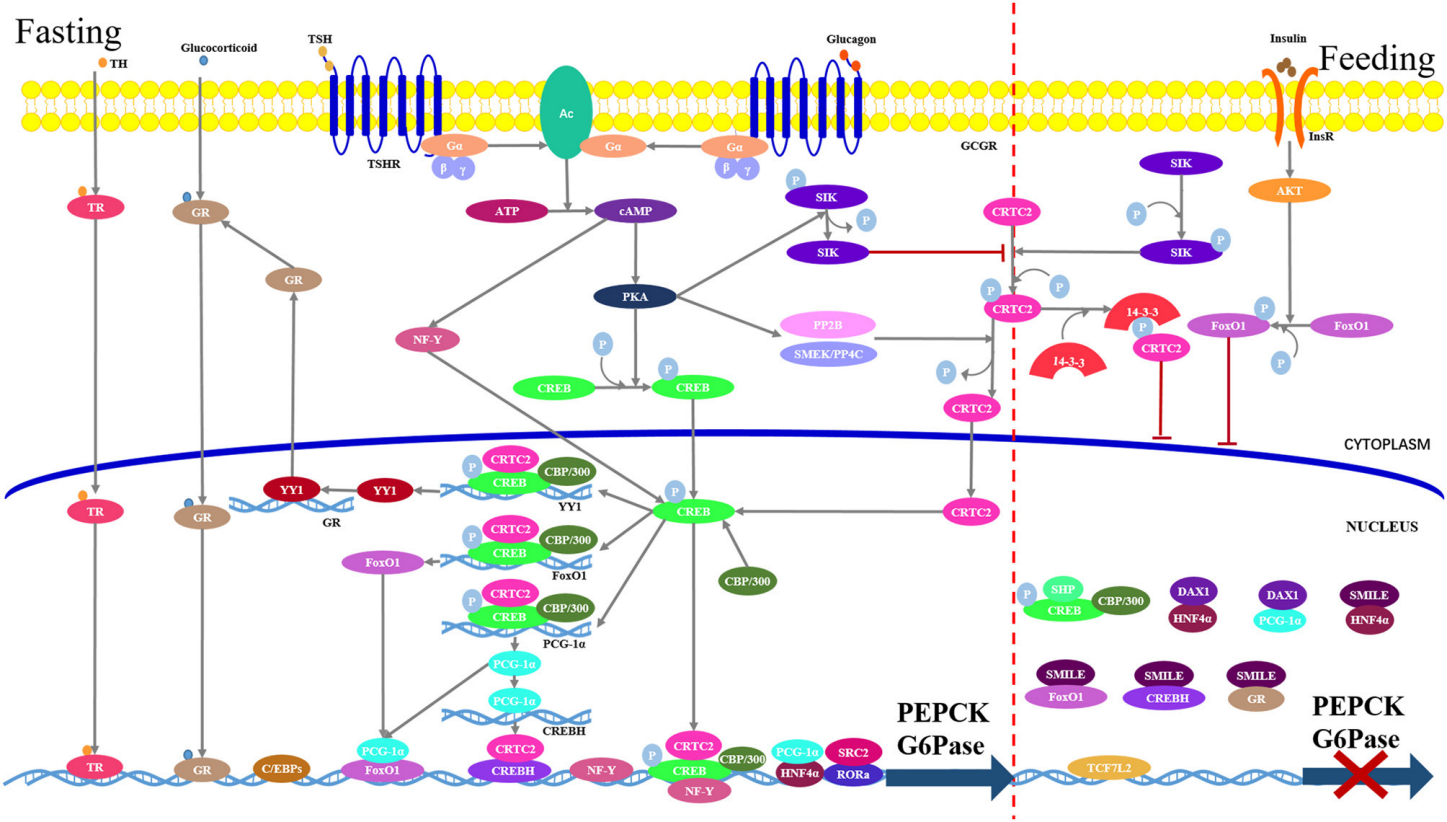
Hepatic gluconeogenesis is regulated by various transcriptional factors in response to the hormonal changes. Under feeding conditions (right part), insulin-dependent activation of the AKT signaling stimulate FoxO1 phosphorylation and cytoplasmic retention. Meanwhile, CREB-regulated transcriptional coactivator-2 (CRTC2) undergoes rapid phosphorylation by salt-inducible kinases (SIKs), which results in its sequestration in the cytoplasm via interaction with 14-3-3 adaptor protein. Transcription factor 7-like 2 (TCF7L2) blocks gene expression through occupying transcriptional recognition sites. Small heterodimer partner-interacting leucine zipper protein (SMILL) and small heterodimer partner (SHP)/DAX1 directly bind to transcriptional factors to interfere with transcription. Under fasting conditions (left part), binding of glucagon or thyroid stimulating hormone (TSH) to its cognate G-protein-coupled receptor, respectively, stimulate adenylyl cyclase (AC), which converts ATP to cAMP. The cAMP in turn stimulates protein kinase A (PKA) to phosphorylate cAMP response element binding protein (CREB). Phosphorylated CREB transfers into the nucleus where it interacts with its coactivator CRTC2 and CBP/300 to form a complex, which binds to the CRE in the gluconeogenic promoters. CREB-CRTC-CBP/300 complex also binds to the promoters of PGC1-1α and FoxO1 and stimulates their transcriptions. Nuclear factor-Y (NF-Y) induced by cAMP interacts with CREB to enhance the expression of PEPCK and G6Pase genes. Glucocorticoid and thyroid hormone (TH) bind gluconeogenic promoters in a hormone-receptor complex manner. Yin Yang 1 (YY1) induced by CREB promotes the GR transcription. C/EBP and HNF4α directly bind to gene promoter. Steroid receptor coactivator 2 (SRC2) functions as a coactivator of receptor-related orphan receptor alpha (RORα), which directly binds to the promoters of PEPCK and G6Pase.

Hepatic gluconeogenesis is hormonally regulated, and numbers of hormones (e.g., glucagon and glucocorticoid) have known stimulatory effects (Figure 2). Of these hormones, glucagon seems to be the most important physiologically. However, only insulin plays a major inhibitory role. The rate of gluconeogenesis is determined by the balance between the stimulatory hormone and the inhibitory one.

## Insulin and Glucagon

Insulin and glucagon are the most important hormones regulating hepatic gluconeogenesis. They demonstrated antagonistic effects on blood glucose levels. Under fasting or feeding, the blood circulating levels of the two hormones will change, subsequently affecting the expression of gluconeogenetic genes.

Insulin is a polypeptide hormone secreted by pancreatic β cells, and its major physiological function is to regulate glucose transport, lipid synthesis, gluconeogenesis, and glycogen synthesis via the PI3K/Akt signaling pathway (13). Under feeding conditions, the raised blood glucose stimulates the secretion of insulin that binds to the insulin receptor (InsR) on the target organ, resulting in activation of InsR kinase and recruitment of insulin receptor substrates (IRS) (14), which in turn recruit and activate PI3K. The activated PI3K rapidly phosphorylates phosphatidylinositol 4,5-bisphosphate (PIP2) to generate the lipid second messenger phosphatidylinositol (3,4,5)-triphosphate (PIP3). The latter recruits AKT (also called protein kinase B, PKB) to the plasma membrane, where it is activated by phosphorylation and regulates glucose utilization through both the FoxO1 and CRTC2. The phosphorylation of FoxO1 and CRTC2 induces their nuclear exclusion and cytoplasmic retention through binding to 14-3-3 protein, suppressing gluconeogenic gene expression.

Glucagon is also a polypeptide hormone secreted by pancreatic α cells, and its primary function is to prevent hypoglycemia. After overnight fasting, the markedly increased glucagon released from the pancreas recognizes and then binds to the hepatic glucagon receptor (GCGR), which is a member of G protein-coupled receptors characterized by being membrane-bound and G protein-coupled (15), forming a glucagon-receptor complex. Subsequently, GCGR undergoes a conformational change that can activate Gsα (G protein). The activated Gsα in turn activates adenylate cyclase that generates the second messenger cAMP, and then cAMP stimulates cAMP-dependent protein kinase (PKA), which phosphorylates the cAMP-response element-binding protein (CREB) (16). Phosphorylated CREB binds to CRE and activates the transcription of CRE-bearing genes (PEPCK and 6Pase gene).

## Glucocorticoid

Glucocorticoid is a kind of sterol hormone secreted by the zona fasciculata of the adrenal glands and its blood levels are under tight regulation by the hypothalamic–pituitary–adrenal axis. It is well-established that glucocorticoid itself can promote hepatic gluconeogenesis. Also, glucocorticoid is important for other hormones (e.g., glucagon) to promote gluconeogenesis in adrenalectomized mice (17). These mice exhibit reduced glucagon or cAMP-induced gluconeogenesis in fasting state, which however can be restored by treating mice with glucocorticoid. In addition, glucocorticoid can promote the generation of gluconeogenic amino acids in skeletal muscle and enhances the release of glycerol in adipose tissue, providing gluconeogenic precursors. On the whole, glucocorticoids play an important role in maintaining glucose homeostasis, its abnormal production results in distinct clinical disease phenotypes, such as Cushing's disease with hyperglycemia and Addison's disease with hypoglycemia (18).

Glucocorticoid functions through binding to intracellular glucocorticoid receptor (GR, also known as NR3C1a), which is a member of the nuclear hormone receptor superfamily. Without ligand binding, GR resides primarily in the cytoplasm in a complex with heat shock proteins (HSP 90 and 70) and co-chaperones Hsp40, p23 and others, leading the GR to preserve a low affinity state (19). Upon ligand binding, GR dissociates from the HSP90 chaperone complex and its DNA binding sites are exposed, then the GR-ligand complex enters the nucleus (20), where the complex either is recruited to specific glucocorticoid-responsive elements (GREs) on gene promoter or interacts with other transcriptional regulators, activating the expression of gluconeogenic genes including PC, PEPCK, FBPase, G6PC, and G6P.

## Hormones in Hypothalamic-Pituitary-Thyroid Axis

Thyrotro-pin-releasing hormone (TRH), secreted from the hypothalamus, acts upon the pituitary gland and binds to G protein-coupled TRH receptors on the thyrotrope, resulting in an increase in intracellular cAMP, and subsequent thyroid-stimulating hormone (TSH) release. Then TSH binds to a G protein-coupled TSH receptor (TSHR) on the thyroid follicular cell, stimulating the production, and release of thyroid hormone (TH), and TH in turn regulates TRH/TSH. This is the fundamental pathway of the hypothalamic-pituitary-thyroid axis.

Studies show that the hormones TSH and TH are closely related to glycometabolism. TSH interacts with its receptor TSHR to activate the PKA via cAMP, PKA in turn inhibits the activation of SIK2 by dephosphorylating at Ser587. The dephospho-CRTC2 enters into the nucleus and interacts with CREB, promoting the expressions of PEPCK and G6Pase genes, and a subsequent increase in hepatic gluconeogenesis (21). In addition, TSH is also associated with hyperinsulinemia and insulin resistance (22). Therefore, the mechanism by which TSH regulates gluconeogenesis is not singular and may involve multiple molecular mechanisms. TH excessively produced by the thyroid gland promotes a hypermetabolic state characterized by increased resting energy expenditure and gluconeogenesis (23). Conversely, the reduced TH levels are associated with hypometabolism characterized by reduced resting energy expenditure and gluconeogenesis. Intracellular triiodothyronine (T3) is the active form of TH and its action is exerted primarily via binding to the TH receptors (TR). TRs are intracellular DNA-binding proteins that can bind to thyroid hormone-response element (TRE) in the promoter regions of target genes. The increased T3 binds to TR across the cell membrane and forms a hormone-TR complex in liver, then the complex directly binds to a TRE to promote the expression of genes encoding the gluconeogenic enzymes PEPCK and P6Gase (24). Additionally, T3-TR complex modulates gluconeogenesis gene expression via some other pathways as it can cross-talk with nuclear receptors (PPARs and LXR), SIRT1, FoxO1, and PCG-1α, which act as transcription factors for gluconeogenesis (24).

## Vasopressin

Vasopressin, also named arginine vasopressin (AVP), is produced in hypothalamus and released by neurohypophysis in a condition of high plasma osmolality, low plasma volume, and low blood pressure (25). AVP is involved in diverse physiological functions, including the regulation of osmotic homeostasis, vasoconstriction, and ACTH release, which are mediated by three types of AVP receptors, designated as V1a, V1b, and V2. AVP also has a role in regulating glycometabolism; it not only stimulates hepatic gluconeogenesis and glycogenolysis by the V1a receptor, but also regulates the secretion of glucagon and insulin by Vlb receptor (26). The V1a receptor triggers Ca^2+^ influx, leading to a decreased concentration of mitochondrial Ca^2+^ and an increased PC activation in hepatocyte. Meanwhile, the Vlb receptor induces Ca^2+^ release from the endoplasmic reticulum (ER), which triggers exocytosis including insulin and glucagon in the pancreas. Under stringent extracellular Ca^2+^ deprivation, AVP still can increase the release of insulin and glucagon; the gluconeogenesis, however, is restrained. This suggests that the action of AVP is sensitive to extracellular Ca^2+^ and may be a Ca^2+^-independent pathway of VAP-induced insulin and glucagon release (27).

## Bile Acids

Bile acids are known mainly as intestinal detergents for the absorption of dietary lipids and fat-soluble vitamins in the gut. In recent studies, bile acids have been proposed to participate in diverse physiologic processes, they also function as hormones or signaling molecules to regulate metabolism of lipids, glucose, and energy expenditure (28). Bile acids accomplish their regulatory functions mainly through two receptors: nuclear farnesoid X receptor (FXR) and G protein-coupled receptor TGR5. It has been revealed that bile acids and FXR upregulate the inhibitory nuclear receptor small heterodimer partner (SHP) in the liver (29, 30). As discussed in a later section, SHP functions as transcriptional co-repressor through interaction with other proteins to repress the transcription of gluconeogenic genes such as PEPCK, G6Pase, and FBPase, resulting in decreased glucose production. In addition, human bile acids act on FXR in ileal enterocytes to induce the expression of fibroblast growth factor (FGF)-19 (its mouse ortholog is FGF-15). FGF15/19 circulates to liver, where it binds to FGF receptor-4 (FGFR4) to inhibit the CREB-PGC-1α signaling cascade, thereby repressing gluconeogenesis (31).

## Transcription Factors Regulates Gluconeogenesis

Hormone release instigates signaling cascades in the liver to modulate activities of various transcription factors, which in turn control the expressions of key rate-limiting enzymes in the gluconeogenic pathway, specifically PEPCK and G6Pase (Figure 2).

## cAMP Response Element Binding protein (CREB)

CREB is a nuclear transcription factor binding to a conserved cAMP-response element (CRE) on gene promoter. CREB can be directly phosphorylated by protein kinase A (PKA) (16). Under fasting conditions, the increased secretion of pancreatic glucagon activates PKA, which in turn phosphorylates CREB, leading to an increased association of CREB with its co-activators CRTC2 and CBP/p300 onto the chromatin. Subsequently, CREB directly drives the gluconeogenic gene transcription by binding to CRE on the promoter and enhances the expression of PEPCK and G6Pase, leading to increased hepatic gluconeogenesis (32). CREB is not only important in the direct transcriptional activation of gluconeogenic genes, but is also critical in modulating the activation of PGC-1α, which serves as a crucial transcriptional regulator for the activation of gluconeogenic genes during prolonged fasting (31). In addition, phosphorylation of CREB-recruiting p300/CBP increases FoxO1 gene expression, which in turn induces the expression of gluconeogenic genes (33).

## Forkhead Box Class O1 (FoxO1)

FoxO proteins belong to a subgroup of the forkhead family of transcription factors that possess a conserved forkhead box-type DNA-binding domain. Four major FoxO proteins (FoxO1, 3, 4, and 6) are identified in mammals, and they are commonly regulated by the insulin/Akt signaling pathway. FOXO transcription factors modulate gene expression in stress resistance, metabolism, apoptosis, and longevity (34). Although FoxO proteins are highly related, only FoxO1 is the most direct transcriptional regulator of gluconeogenesis (35). During fasting state, FoxO1 is activated by dephosphorylation and translocated into the nucleus, leading to the transcriptional induction of G6Pase and PEPCK and an increased production of hepatic glucose (36). During feeding state, FoxO1 is negatively inhibited by the insulin signaling pathway. The increased secretion of pancreatic insulin activates PI3 kinase and the subsequent Akt, which in turn phosphorylates FoxO1 at Thr24, Ser253, and Ser316, and phosphorylation of FoxO1 exports from the nucleus, leading to a decreased occupancy of FoxO1 over the promoters of gluconeogenic genes and an attenuated gluconeogenesis (37). In the process of FoxO1 regulating gluconeogenesis, PGC-1α, and protein arginine methyltransferases 1 (PRMT1) act as co-activators and are crucial for the transcription of gluconeogenic genes (38, 39).

## cAMP Response Element-Binding Protein H (CREBH)

CREBH belongs to ER-bound transcription factor families, and it is mainly expressed in the liver. CREBH was recently identified as an important physiological regulator of hepatic gluconeogenesis (40). During fasting or insulin-resistant state, the expression of CREBH is markedly induced by the PGC-1α/GR complex, resulting in the accumulation of CREBH-N, which is an active nuclear form of CREBH. By cross-talking with its co-activator CRTC2, CREBH-N enhances hepatic gluconeogenesis by binding to a unique regulatory sequence in the promoter of the PEPCK or G6Pase genes (41). One recent study shows that the proteolytic cleavage and posttranslational acetylation modification of CREBH are regulated by the circadian clock (40), by which CREBH can regulate the rhythmic expression of the genes encoding the rate-limiting enzymes for glycogenolysis and gluconeogenesis, including liver glycogen phosphorylase, PEPCK, and G6Pase. In addition, the acetylation of CREBH at lys-294 controls CREBH-PPARα interaction and synergy in regulating hepatic glucose metabolism in mice (40).

## CCAAT/Enhancer-Binding Proteins (C/EBPs)

C/EBPs are key regulators of numerous cellular processes, including cell proliferation, differentiation, tumorigenesis, and gluconeogenesis and lipid synthesis (42). All C/EBPs (C/EBPα, β, δ, ε, and γ) consist of an N-terminal transactivation domain (TAD), central regulatory regions, and a highly conserved C-terminus basic region leucine zipper (bZIP) domain. Targeted deletion of the C/EBPα gene in mice results in profound damage to liver structure and function and postnatal death because of hypoglycemia caused by defects in the hepatic gluconeogenesis and glycogen storage (43). Studies have shown that C/EBPα can be phosphorylated by p38 at serine 21, and the phosphorylated C/EBPα exhibits increased transactivation activity to enhance PEPCK gene transcription (44). C/EBPβ can also bind to PEPCK promoter directly to activate gene expression, and C/EBPβ deficient mice show impaired hepatic glucose production (45). C/EBPβ activity is negatively regulated by AMP-activated protein kinase (AMPK), where activated AMPK suppresses C/EBPβ expression and inhibits PEPCK transcription in diabetes (46).

## Nuclear Receptors (NRs)

NRs are a superfamily of transcription factors. A typical nuclear receptor consists of a highly variable NH2-terminal A/B domain that contains a transactivation function known as AF-1, a central conserved DNA binding domain (DBD or C domain), a short D domain that contains the nuclear location signal, a large conserved carboxyl-terminal E domain (LBD) that is responsible for the binding of cognate ligand or hormone and contains ligand-regulated transcriptional activation function 2 (AF-2) (47). NRs usually bind directly to the response elements of genes and regulate gene transcription. NRs exist as monomers, homodimers, or heterodimers.

Homodimeric NRs, such as glucocorticoid receptor (GR) and hepatocyte nuclear factor 4α (HNF4α), normally reside in the cytosol via interaction with chaperon proteins (e.g., HSP90 and HSP70). Upon binding ligand, the receptor disassociates from the chaperone and homodimerize, leading to the exposure of nuclear localization sequence and entry into the nucleus. HNF4α is hepatocyte-specific transcription factor mainly regulating hepatic differentiation (48), which is demonstrated to promote the expression of PEPCK and G6Pase via binding to the cis-elements in their promoters (49). TR4 can regulate the expression of gluconeogenic genes via binding to its novel responsive element (TR4RE) in the gene 5′-flanking region (50). Elimination of TR4 in hepatocytes significantly reduces PEPCK gene expression and glucose production in response to glucose depletion, while ectopic expression of TR4 increases PEPCK expression and hepatic glucose production in human and mouse hepatoma cells (33). NR4A contains three closely related receptors (Nur77, Nurr1, and NOR1), which are induced by multiple extracellular signals, including growth factors, apoptotic, and inflammatory signals and hormones in a cell type-specific manner (51). NR4A receptors not only are responsive to cAMP second messenger pathways in muscle and pituitary, but also are downstream mediators of the glucagon-cAMP-CREB axis in the control of glucose metabolism in liver (52).

Heterodimeric NRs (e.g., retinoic acid receptor, thyroid hormone-receptor, and the peroxisome proliferator-activated receptor α, PPARα) reside in the nucleus and form heterodimers with retinoid X receptor (RXR), which is generally bound to the target promoter through interactions with corepressor complexes NCoR and SMRT in the absence of ligand. Ligand binding results in the replacement of corepressors with coactivator complexes and facilitates gene transcription. PPARα, highly expressed in liver and brown adipose tissue, recognizes, and binds to specific DNA sequences AGGTCA (PPAR response elements) with the RXR. PPARα has been identified as a direct activator of numerous gluconeogenic genes (53). Glucagon increases hepatic PPARα activity, while insulin- and nutrient-sensitive pathways repress PPARα activity through its co-repressor NCoR1 (54).

Monomeric NRs, such as retinoic acid receptor-related orphan receptor alpha (RORα), bind as monomers to half-site response elements which typically consist of a consensus AGGTCA half-site. RORα has been shown to directly bind to the promoters of PEPCK and G6Pase and regulates their transcriptions. Mice treated with RORα antagonists or targeted deletion of the RORα gene exhibit decreased PEPCK expression and glucose production. Similarly, the expression of G6Pase and PEPCK is suppressed in HepG2 cells overexpressing Rev-erbα, the physiological repressor of RORα. In contrast, silencing Rev-erbα significantly promotes G6Pase and PEPCK expression. Furthermore, there is evidence that steroid receptor coactivator 2 (SRC2) functions as a coactivator of RORα to enhance the rate of transcription of G6Pase; both RORα and SRC2 are required for the stimulatory effect of PGC-1α on G6Pase transcription.

## Other Transcriptional Factors

Yin Yang 1 (YY1), belonging to the polycomb group protein family, is widely expressed in various tissues. YY1 binds to CCATNTT consensus sequences to activate or silence gene transcription via chromatin modification. During states of fasting or insulin resistance, YY1 transcriptionally upregulates GR expression, which then enhances the expression of gluconeogenic enzymes PEPCK and G6Pase, promoting hepatic glucose production (55).

Nuclear factor-Y (NF-Y) is a trimeric protein complex composed by NF-YA, NF-YB, and NF-YC subunits, and it can specifically recognize and bind the CCAAT box. We recently observed that the hepatic NF-Y expression is markedly induced by cAMP, glucagon and fasting, and knockdown of NF-YA weakens gluconeogenic gene expression and glucose production in mice. NF-Y enhances the expressions of PEPCK and G6Pase either by binding to their promoters directly or interacting with CREB (56).

Transcription factor 7-like 2 (TCF7L2, also known as TCF4) is demonstrated as a strong candidate for type 2 diabetes in recent extensive genome-wide association studies (57). TCF7L2 plays a crucial role in suppressing hepatic gluconeogenesis. In the fed state, increased TCF7L2 binds to the promoters of the genes PEPCK and G6Pase, interfering the occupancy of both CREB and FoxO1 on their chromatin recognition sites (58). Under insulin-resistant conditions, the decreased expression of TCF7L2 in the nucleus may fail to inhibit CREB/FoxO1-dependent gluconeogenesis (59). In addition, TCF7L2 mediates glucose homeostasis by enhancing glucagon-like peptide-1 (GLP-1) secretion and GLP-1-dependent insulin secretion (60).

## Cofactors Regulating Transcription in Gluconeogenesis

Transcriptional cofactors regulate gluconeogenesis by directly binding to transcriptional factors to either activate or inhibit transcriptions (Figure 2).

## PPARγ Coactivator 1-alpha (PGC-1α)

PGC-1α is a transcriptional coactivator that involves the regulation of energy metabolism. The expression of PGC-1α is markedly induced in the liver upon prolonged fasting and in multiple diabetes animal models (61). Under fasting conditions, the increased PGC-1α interacts with various gluconeogenic transcription factors such as FoxO1, GR, and HNF4α, enhancing their transactivating potentials and promoting the expression of gluconeogenic genes (62). Liver-specific PGC-1α gene deletion studies (63) and acute RNAi-based PGC-1α hepatic knockdown strategies (64) in mice lead to diminished expression of genes encoding gluconeogenic enzymes.

## cAMP-Regulated Transcriptional Coactivators (CRTC2)

CRTC2 is a member of the CRTC transcriptional coactivators family known as transducers of regulated CREB activity (TORC). There are three CRTC isoforms (CRTC1, 2, and 3) in mammals: CRTC1 is expressed predominantly in the brain, CRTC3 is expressed highly in adipose tissue, whereas CRTC2 is found mainly in liver. CRTCs contain an N-terminal CREB-binding domain, a central regulatory domain, and a negatively charged C-terminus TAD. The activity and cellular localization of CRTCs are phosphorylation-dependently regulated. Under feeding, liver kinase B1 (LKB1) constitutively activates salt-inducible kinases (SIKs) through phosphorylation of a threonine residue in the activation loop of their kinase domain, leading to the subsequent phosphorylation of CRTCs (65), and phosphorylated CRTCs can bind to 14-3-3 protein and reside in the cytoplasm. Under fasting conditions, the activated serine/threonine phosphatases (e.g., PP2B or SMEK/PP4C) lead to rapid CRTC2 dephosphorylation. The dephosphorylated CRTC2 shuttles to the nucleus and interacts with chromatin-bound CREB, triggering gluconeogenic gene expression (66). Dephosphorylated CRTC2 also promotes increased histone H3 acetylation at Lys 9 (H3K9Ac), which subsequently potentiates CRTC2 occupancy over promoters of PEPCK, G6Pase, or PGC-1α (67). In mammals, CRTC2 is also shown to directly bind to and coactivate additional bZIP transcription factors, such as CREBH and ATF6, regulating glucose homeostasis (41). Indeed, targeted deletion of the CRTC2 gene in mice attenuates the expression of gluconeogenic enzyme genes, resulting in a reduction in circulating blood glucose levels (68).

## P300 and CREB Binding Protein (CBP)

P300 and CBP are histone acetyltransferases transferring an acetyl group to lysine residue, and the acetylation level of non-histone proteins has been identified as a key mechanism for regulating hepatic gluconeogenic gene transcription (69). P300/CBP is implicated in glucose metabolism due to its role as a transcriptional coactivator for CREB. Additionally, P300/CBP can promote FoxO1 expression by binding to the CREs in the proximal promoter region (70).

## Small Heterodimer Partner-Interacting Leucine Zipper Protein (SMILE)

SMILE is a member of the CREB/ATF (activating transcription factor) family of bZIP transcription factors. Under feeding or insulin-resistant conditions, insulin can promote hepatic SMILE expression (71). SMILE competitively binds to nuclear receptors, such as estrogen-related receptor γ, CREBH, GR, constitutive androstane receptor, and HNF4α, and represses their transcriptional activities. SMILE can decrease the stimulatory effect of CREB/CRTC2 on hepatic gluconeogenesis (71).

## DAX-1 and Small Heterodimer Partner (SHP)

DAX-1(also known as NR0B1) and SHP (also known as NR0B2) are members of an orphan nuclear receptor family of unknown endogenous ligands. However, unlike other nuclear receptors, both DAX and SHP lack a DNA-binding motif, and they primarily function as transcriptional corepressors through interaction with other proteins (72). SHP is predominantly found in the liver and represses the transcriptional activities of various nuclear receptors by inhibiting their occupancy over the cognate response elements in the promoters, such as GR, estrogen receptor, androgen receptor, steroidogenic factor 1, and HNF4α. In addition, SHP can directly bind to CREB to block the association with CRTC2, leading to an inhibition of hepatic gluconeogenic gene expression (73). DAX-1 inhibits transcription via its LXXLL motifs and is mainly involved in steroidogenesis and reproductive development (72). There is evidence that DAX-1 can reduce hepatic gluconeogenesis by inhibiting the recruitment of other transcription factors or coactivators, such as HNF4α and PGC-1α onto the promoters of PEPCK and G6Pase (74).

## Post-Translational Modifications (PTMs) Modulate Gluconeogenesis

PTMs including phosphorylation, (de)methylation, (de)acetylation, ADP-ribosylation and ubiquitination, and modulate gene transcription in a cell- and tissue-specific manner. The role of PTMs in the regulation of gluconeogenesis has received close attention. The aforementioned phosphorylation of transcriptional activators, such as FoxO1, CREB, and CRTC2, exemplifies a typical mechanism whereby PTMs directly regulate gluconeogenesis. In addition, Lysine acetylation and arginine methylation have been identified as evolutionarily conserved PTMs in gluconeogenic regulators and have emerged to play major roles in mediating gluconeogenesis.

## Phosphorylation by AMP-Activated Protein Kinase (AMPK)

AMPK is an evolutionarily conserved serine/threonine heterotrimeric protein kinase, which consists of a catalytic α-subunit and two regulatory subunits, β-subunits and γ-subunits. AMPK is activated when cellular energy levels are low, switching off ATP-consuming anabolic pathways and switching on ATP-generating catabolic processes. It has been reported that acute hepatic overexpression of a constitutively active form of AMPKα2 suppresses the expression of key hepatic gluconeogenic genes, G6Pase, and PEPCK, resulting in a depressed hepatic glucose output (75). Compound A-769662 is identified as a small-molecule AMPK activator, which has a robust anti-diabetic effect in ob/ob mice by impacting liver gluconeogenic pathways (76). AMPK can control gluconeogenic gene expression by promoting CRTC2 phosphorylation (77), and phosphorylated CTRC2 is sequestered in the cytoplasm and inhibits CREB-dependent transcriptions of PGC1α and gluconeogenic enzyme genes. In addition, as discussed in a later section, AMPK phosphorylates and nuclear exclude class II histone deacetylases (HDACs) that modulate the transcription of gluconeogenic genes (78).

Although many studies reported that AMPK acts as a regulator of hepatic gluconeogenesis, they have been performed by using non-specific AMPK activators or pharmacological compounds with AMPK-independent side effects. In addition to being an AMPK activator, A-769662 is found to inhibit Na+-K+-ATPase in L6 skeletal muscle cells or non-proteolytic components of the 26S proteasome by an AMPK-independent mechanism (79, 80). Moreover, a rich body of literature suggests that AMPK might not function as a regulator of hepatic gluconeogenesis (81–83), such as with compound PF-739, which is an AMPK-specific activator but has no impact on the endogenous glucose production rate in *in vivo* clamp studies (82). Therefore, the role of AMPK in the regulation of gluconeogenesis remains uncertain.

## Methylation by Protein Arginine Methyltransferases (PRMTs)

**Figure 3:**
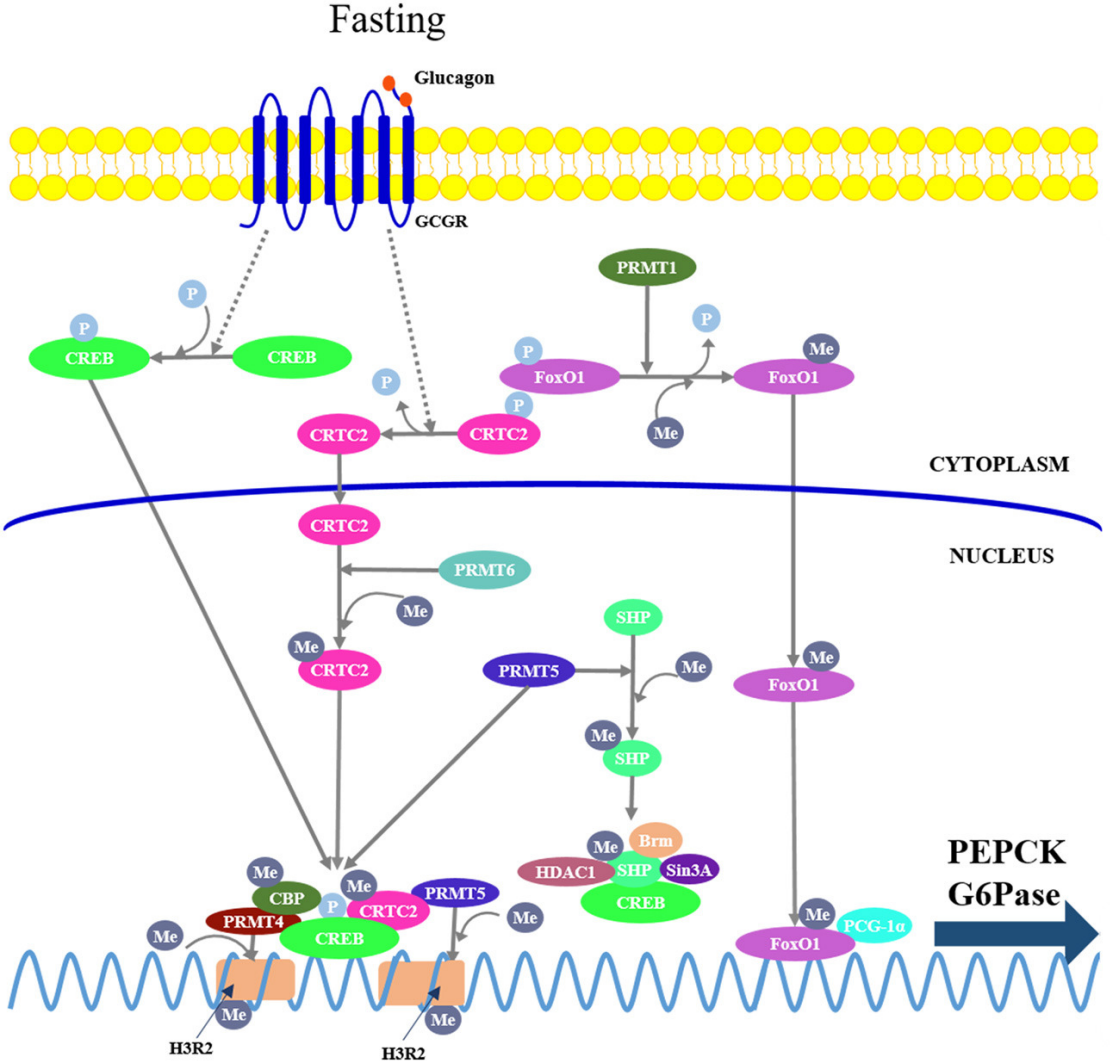
Protein arginine methyltransferases (PRMTs) mediate hepatic gluconeogenesis. PRMT1 catalyzes asymmetric methylation of FoxO1, repressing phosphorylation, and promoting its nuclear translocation. PRMT4 and 5 are recruited to the gluconeogenic promoters by interacting with CREB and CRTC2, respectively, where they mediate symmetric dimethylation of histone H3 at arginine 2 (H3R2), leading to enhanced chromatin accessibility at promoters and promoting CREB-dependent transcription. On the other hand, PRMT4 also can mediate arginine methylation in the KIX domain of CBP, leading its dissociation from CREB; PRMT5 can directly promote arginine methylation of SHP, which in turn recruits other chromatin-modifying repressive cofactors (Brm, Sin3A, and HDAC1) to repress gluconeogenic gene expression. PRMT6 can directly mediate asymmetric dimethylation of CTRC2 to enhance the interaction between CRTC2 and CREB.

PRMTs catalyze the transfer of methyl groups to arginine, and nine isoforms (PRMT1-9) consisting of three major types (type I-III) have been identified in mammals (84). Type I PRMTs including PRMT1, 3, 4, 6, and 8 usually function as transcriptional activators by promoting asymmetric dimethylation of arginine on histones or transcriptional regulators. Type II PRMTs comprising PRMT5, 7, and 9 generally function as transcriptional repressors by mediating symmetric dimethylation of arginine on histone. Type III PRMTs are less well-characterized in the control of transcription. Recent reports suggest that PRMT1, 4, 5, and 6 can control hepatic gluconeogenesis through specific modulation of FoxO1- and CREB-dependent transcriptional events (Figure 3).

PRMT1 catalyzes asymmetric methylation of FoxO1 at arginines 248 and 250, thereby repressing phosphorylation of the adjacent serine 253 (85), promoting its nuclear translocation and enhancing its chromatin occupancy over the promoters of gluconeogenic genes. Either acute knockdown or chronic haploinsufficiency of PRMT1 in mice reduces FoxO1-mediated gluconeogenesis, indicating that PRMT1 is critical in enhancing FoxO1 activity in the physiological context (86).

PRMT4, also known as coactivator-associated arginine methyltransferase 1, modulates hepatic gluconeogenesis through interaction with CREB in hepatocytes. PRMT4 can mediate asymmetric dimethylation of histone H3 at arginine 2 (H3R2), thereby promoting its acetylation by CBP/p300, leading to an increased CREB-dependent transcription of gluconeogenic genes (87). However, there is evidence that PRMT4 can mediate arginine methylation in the KIX domain of CBP, leading its dissociation from CREB, resulting in an inhibition of CREB-dependent transcription (88). Therefore, PRMT4-dependent regulation of gluconeogenesis requires further study.

PRMT5 regulates gluconeogenesis either as a coactivator of CREB or a corepressor of SHP. In fasting conditions, the elevated glucagon promotes the interaction of PRMT5 with CRTC2, by which PRMT5 is recruited to the gluconeogenic promoters and mediates symmetric dimethylation of H3R2, leading to enhanced chromatin accessibility at promoters, stimulating CREB phosphorylation, and gluconeogenic gene expression (89). In addition, PRMT5 can directly promote methylation of SHP at Arg57, which in turn recruits other chromatin-modifying repressive cofactors, such as Brm, Sin3A, G9a, and HDAC1, to repress gluconeogenic gene expression (90). Thus, further study is required to delineate whether the SHP activation compromises the CREB-dependent transcription.

PRMT6 can directly mediate asymmetric dimethylation of CTRC2 at multiple arginine residues, resulting in an enhanced interaction between CRTC2 and CREB on gluconeogenic promoters (91).

## Histone Demethylation by Demethylases

Histone methylation, occurring on specific lysine and arginine residues in histones H3 and H4, has been associated with activated or repressed transcription. Lysine residues on histone tails can be mono-, di-, or trimethylated, while arginine residues can be mono and asymmetrically or symmetrically di-methylated. The site and degree of histone tail methylation have a distinct effect on gene regulation. For instance, histone H3 lysine-4 (H3K4) and H3K36 di- or trimethylation (Me2 or Me3) and H3K27 mono-methylation are positively correlated with transcriptional activity, whereas H3K9 and H3K27 Me2 and Me3 are found to mark the repressed genes (92).

Histone methylation is a dynamic and reversible process controlled by a balance between histone methyl-transferases and demethylases. Since the amine oxidase LSD1 (KDM1) is identified as the first histone lysine-specific demethylase, a number of histone demethylases have been identified, such as H3K9-specific demethylase JHDM2A (KDM3A), H3K27-specific demethylases UTX (KDM6A), and JMJD3 (KDM6B) (93). LSD1 histone demethylases require flavin adenine dinucleotide (FAD) as a co-factor (94). JHDM2A, UTX, and JMJD3 contain a JmjC domain that possesses demethylation activity, and these enzymes use Fe(II) and the intermediate metabolite a-ketoglutarate as co-factors to catalyze a hydroxylation-based demethylation (95).

Accumulated evidence has suggested that histone demethylation suppresses the expression of gluconeogenic genes and glucose production. For example, in one study in H4IIE rat hepatoma cells, Hall et al. observed that insulin induces demethylation of arginine-17 on histone H3 at promoters of PEPCK and G6Pase, which correlated with the disruption of the PEPCK and G6Pase gene transcription complex, resulting in the repression of gene transcription (96). In a study from Pan et al. H3K36 demethylase JHDM1a (KDM2A) was identified as a negative transcriptional regulator of gluconeogenesis (97). The authors demonstrated that the regulation of gluconeogenesis by JHDM1a requires demethylation activity, and JHDM1a actively removes dimethyl groups from histone H3K36 on the C/EBPa locus and negatively regulates its expression, which in turn results in a decreased expression of gluconeogenic genes. In a recent study on human HepG2 cells, Pan et al. again reported that knockdown or pharmacological inhibition of LSD1 caused an increase of H3K4 dimethylation levels at promoters of FBP1 and G6Pase and an upregulation of their expression, leading to enhanced gluconeogenesis, and diminished intracellular glycogen content (98).

## Acetylation by Histone Acetyltransferases (HAT) and Deacetylation by Histone Deacetylases (HDACs)

Acetylation of histone by HAT reduces the affinity between histone and DNA and allows transcription factors more accessible to DNA. Initially, acetylation is primarily identified on histone or chromatin-associated factors. Recent studies have shown that many transcriptional regulators are modulated by acetylation on lysine residues or that they are themselves acetyltransferase (99, 100). Therefore, protein acetylation plays a fundamental role in the regulation of gene expression. For example, in addition to being a CREB coactivator, P300 functions as PEPCK acetyltransferase to modulate gluconeogenesis. High-concentration glucose induces P300-catalyzed PEPCK acetylation, which stimulates its interaction with the E3 ubiquitin ligase UBR5, which ubiquitinates the acetylated PEPCK, leading to PEPCK degradation in a proteasome-dependent manner (101). As a result, gluconeogenesis is suppressed.

**Figure 4:**
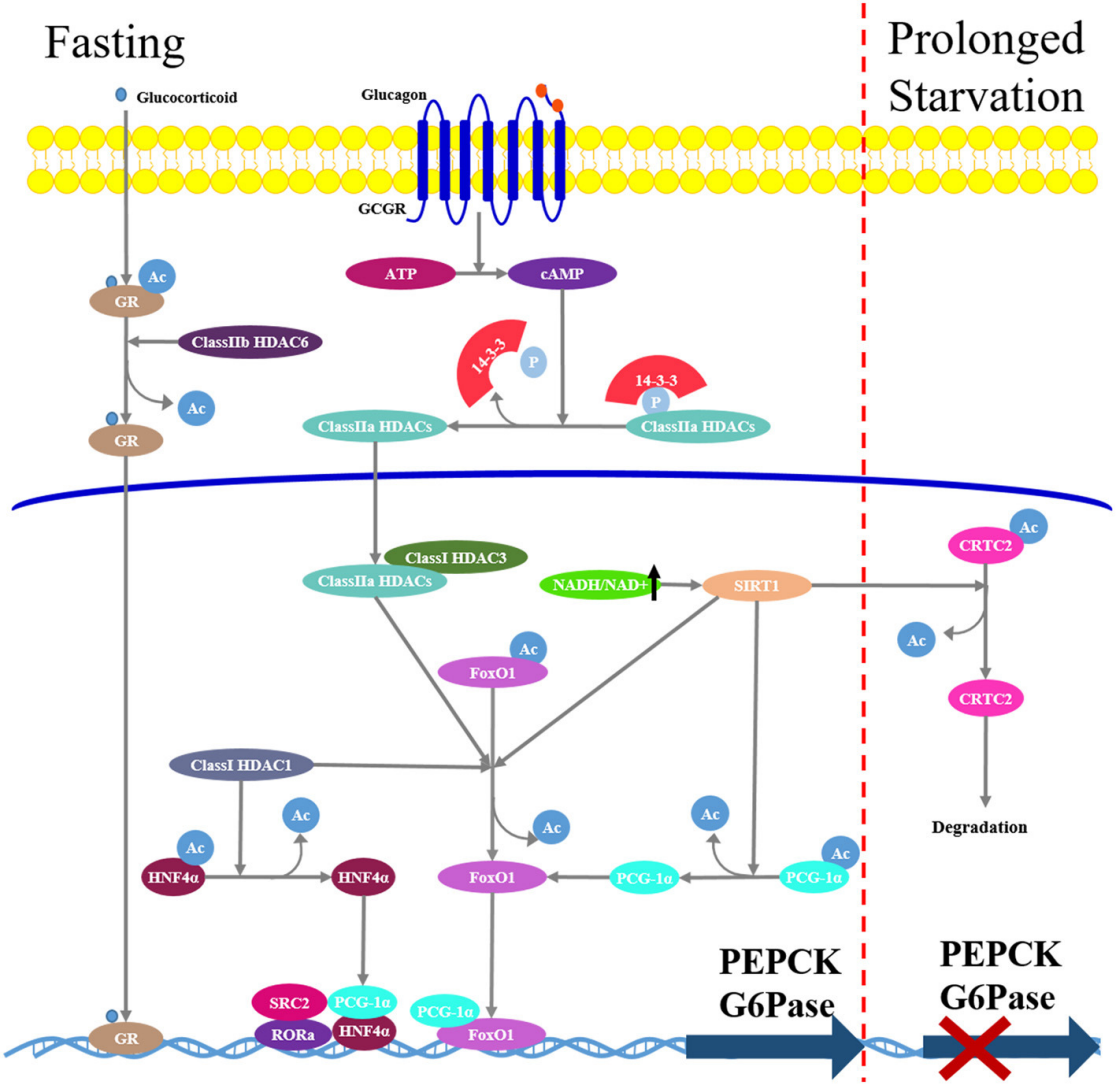
Hepatic gluconeogenesis is modulated by histone deacetylases (HDACs). In fasting state, increased glucagon/cAMP/PKA signaling pathway induces class IIa HDACs dephosphorylation and nuclear translocation. In nucleus, they recruit class I HDAC3 and form a complex that promotes deacetylation and activation of FoxO1. Class IIb HDAC6 promotes glucocorticoid deacetylation and nuclear translocation. Class III HDACs SIRT1 mediates deacetylation and activation FoxO1 and PGC-1α. During prolonged starvation, Sirt1 can repress glucagon-induced gluconeogenesis through deacetylation and subsequent ubiquitin-dependent degradation of CRTC2.

In contrast to HAT, HDACs remove acetyl groups, resulting in chromatin remodeling and transcription inhibition. In mammals, there are eighteen HDACs and they are divided into four classes (class I-IV) based on their sequence identity and catalytic activity (102). Class I HDACs (HDAC1-3 and 8) are yeast reduced potassium deficiency 3 (Rpd3)-like enzymes localized in the nucleus; Class II HDACs are yeast Hda1 protein-like enzymes subdivided into class IIa (HDAC4, 5, 7, and 9) and IIb (HDAC6 and 10); Class III HDACs are yeast silent information regulator 2 (Sir2)-like enzymes and therefore are also called sirtuins (SIRTs), which are nicotinamide adenine dinucleotide (NAD)-dependent deacetylases. In mammals, there are seven sirtuins (SIRT1-7) localized in different subcellular regions. SIRT1 and 2 are found in both the nucleus and the cytoplasm, SIRT 3-5 are found predominantly in the mitochondria, whereas SIRT6 and 7 are localized mainly in the nucleus. Class IV includes HDAC11 localized in the nucleus. Numerous studies have demonstrated that HDACs are extensively involved into the regulation of glucose metabolism by controlling the activities of various gluconeogenic transcriptional factors (103) (Figure 4).

Class I HDACs increase the expression of nuclear receptor HNF4α and the transcriptional activity of FoxO1 in hepatocytes, inducing PEPCK expression, and hepatic gluconeogenesis (104).

Class IIa HDACs play an important role in the regulation of hormone-induced FoxO (78). Under feeding conditions or metformin treatment, activated insulin/Akt, or LKB1-AMPK signaling pathways induce phosphorylation of class IIa HDAC4/5/7 at Ser259 and Ser498, phosphorylated HDACs are excluded from the nucleus and bind to 14-3-3 adapter protein and reside in the cytoplasm. Under fasting conditions, an increased glucagon/cAMP/PKA signaling pathway induces class IIa HDAC dephosphorylation and nuclear translocation. In the nucleus, class IIa HDACs recruit Class I HDAC3 and form a complex that promotes FoxO1 deacetylation, enhancing FoxO1 DNA-binding and transcriptional induction of gluconeogenic genes (78). Indeed, similar to targeted hepatic FoxO1 deficiency in mice, loss of class IIa HDACs in liver results in lowered blood glucose and increased glycogen storage (105). In addition, class IIb HDAC6 is recently demonstrated to play an important role in glucocorticoid-mediated gluconeogenesis (106). HDAC6 deficiency in mice attenuates GR nuclear translocation, which is required for glucocorticoid response in relation to hepatic gene expression, resulting in a diminished expression of glucocorticoid-induced gluconeogenic genes and decreased hepatic glucose production.

Among Class III HDACs, SIRT1 is the most studied in gluconeogenesis. During fasting, SIRT1 mediates deacetylation and activation of transcription factors FoxO1 and PGC-1α, resulting in an elevated expression of gluconeogenic genes and hepatic glucose output (107). Additionally, SIRT1 has been shown to deacetylate and inactivate the hepatic signal transducer and activator of transcription-3 (STAT3) (108), which is acetylated by HAT P300 and in turn suppresses gluconeogenic enzyme expression. Accordingly, inhibition of SIRT1 expression in diabetic rats reduced blood glucose concentrations and hepatic expression of gluconeogenic genes as a result of increased acetylation of FoxO1, PGC-1α, and STAT3 (109). On the other hand, SIRT1 can downregulate hepatic expression of gluconeogenic genes and reduce gluconeogenesis during prolonged starvation; this is attributed to the deacetylation and subsequent ubiquitin-dependent degradation of CRTC2 (110). Additionally, there is evidence that metformin-induced AMPK activation increases the NAD+/NADH ratio and SIRT1 activity (111, 112), resulting in a reduction of gluconeogenesis in the liver.

Additionally, SIRT2, SIRT3, SIRT6, and SIRT7 have been implicated as potential regulators of blood glucose metabolism (101, 113–115). Therefore, sirtuins are promising pharmacological therapeutic targets for the treatment of insulin resistance and subsequent T2DM.

## Hepatic Gluconeogenesis Is a Target of Metformin

**Figure 5:**
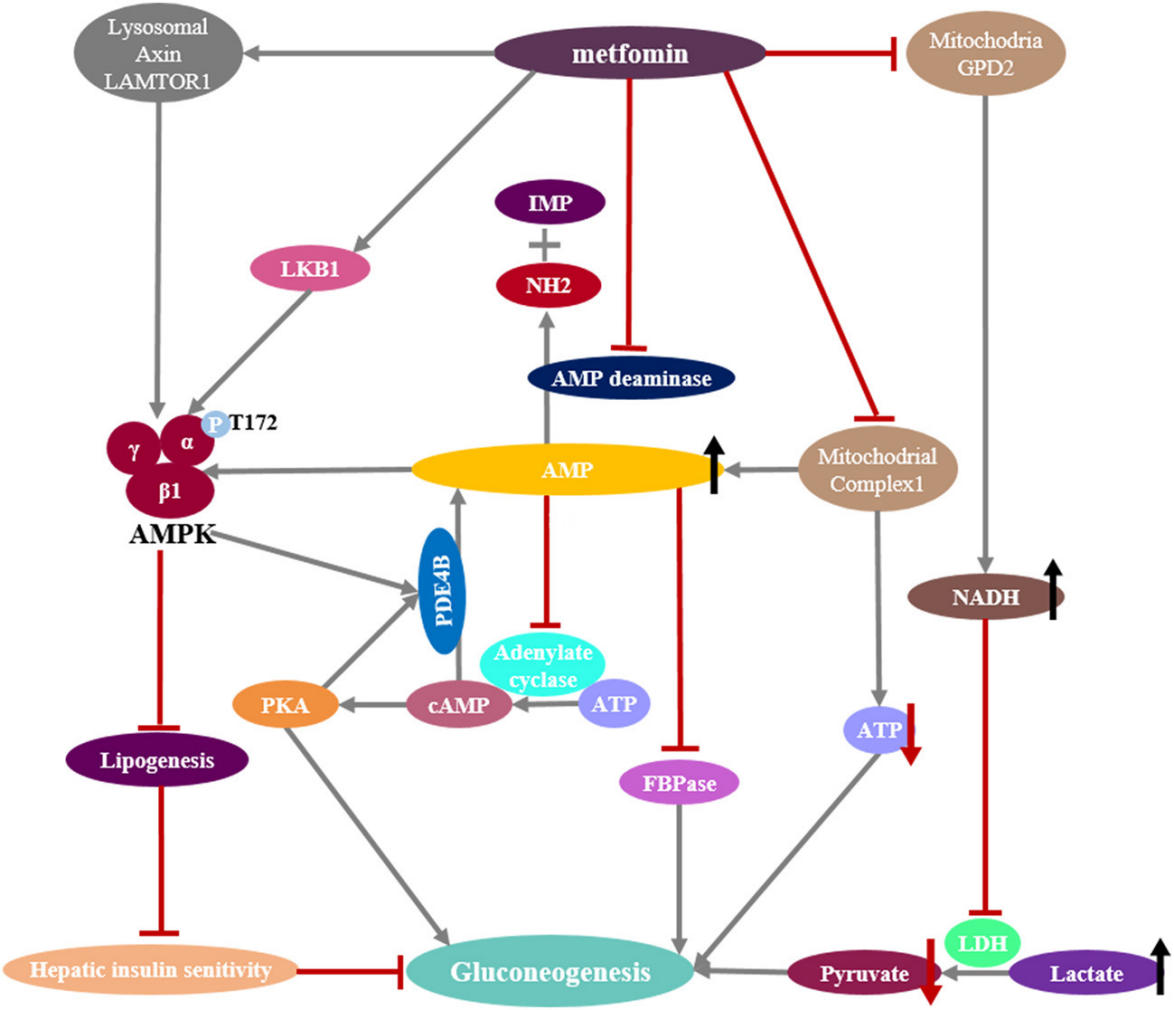
Mechanisms of metformin suppressing hepatic gluconeogenesis. **(Left)** Pharmacologic metformin concentrations activates AMPK by promoting the formation of the AMPK heterotrimeric complex or the phosphorylation of serine/threonine kinase 11 (STK11/LKB1) or through a lysosomal mechanism requiring Axin and late endosomal/lysosomal adaptor, MAPK and mTOR activator 1 (LAMTOR1). Activated AMPK reduces hepatic lipogenesis and increases insulin sensitivity. Activated AMPK also phosphorylates and activates the cAMP-specific phosphodiesterase 4B (PDE4B), which triggers cAMP breakdown. **(Middle)** Supra-pharmacologic metformin concentrations inhibit mitochondrial complex I, as does the inhibition of AMP deaminase, preventing mitochondrial ATP production and increasing cytoplasmic AMP levels. Reduction of cellular ATP levels leads to the suppression of hepatic gluconeogenesis that is an energy demanding process. Elevated AMP levels not only block the cAMP-PKA pathway by the inhibition of adenylyl cyclase activity, also inhibit the gluconeogenic rate-controlling enzyme FBPase and activate AMPK. **(Right)** Metformin inhibits mitochondrial glycerol 3-phosphate dehydrogenase (G3PDH, also named GPD2), resulting in an increase in cytosolic NADH levels and a suppression of lactate utilization and a consequent decreased gluconeogenesis.

Metformin, a primary hypoglycemic agent for type 2 diabetes, ameliorates hyperglycemia predominantly through suppression of hepatic gluconeogenesis (116). Several molecular mechanisms have been proposed to be metformin's mode of action (Figure 5).

## Metformin Suppresses Hepatic Gluconeogenesis in AMPK-Dependent Ways

Metformin-induced modulation of AMPK activity has been previously considered to be central to its physiological functions, including the inhibition of gluconeogenesis (117, 118). Low metformin concentrations (≤ 80 μM) can promote the phosphorylation of serine/threonine kinase 11 (STK11/LKB1), which in turn phosphorylates T172 on α1 subunit of AMPK and activates AMPK (119, 120), and genetic ablation of liver LKB1 eliminates the ability of metformin to activate AMPK *in vivo*. There is evidence that metformin could promote the assembly of a functional AMPK_αβγ_ heterotrimeric complex, making it more easily phosphorylated at T172 of subunit α1 by upstream kinase LKB1, as well as hindering the dephosphorylation of the T172 by protein phosphatase (121). More recent evidence suggests that metformin can activate AMPK through promoting the formation of the vascular ATPase-Ragulator-AXIN/LKB1-AMPK complex on the lysosome surface independent of energy state (122). It has been proposed that metformin-activated AMPK leads to phosphorylation-induced activation of the cyclic nucleotide phosphodiesterase 4B (PDE4B), which triggers cAMP breakdown to reduce the glucagon-stimulated rise in cAMP and PKA signaling (123). However, the role of AMPK in mediating the anti-hyperglycemic action of metformin remains controversial (81). It has been demonstrated that AMPK is neither sufficient nor necessary for acute inhibition of hepatic glucose production, which is supported by the observation that mice ablating AMPK catalytic subunits in the liver display blood glucose levels comparable with those of wild-type mice (81). Of significance, the repression of G6Pase expression in response to metformin treatment is preserved in mouse primary hepatocytes in which AMPK or LKB1 had been depleted (81). In order to help to reconciliate ongoing controversy and unify apparently conflicting data (81, 118), it has been hypothesized that AMPK might play an indirect role in the inhibition of hepatic glucose production as a consequence of AMPK-induced improvement of hepatic insulin sensitivity in obese diabetic mice (81, 124). AMPK is known as a critical regulator of lipogenesis (125), and chronic treatment of obese diabetic mice with metformin profoundly reduces hepatic steatosis and improves insulin resistance (126). Therefore, the glucose-lowering effect of metformin in obese diabetic mice might be due to the impact of AMPK activation on the reduction of hepatic fat content but not gluconeogenesis, leading to a subsequent increase in hepatic insulin sensitivity and ultimately to the normalization of blood glucose levels.

## Metformin Suppresses Hepatic Gluconeogenesis in AMPK-Independent ways

Mechanistic studies suggest that the primary site of metformin action appears to be the respiratory chain complex I (Figure 5), which catalyzes the transfer of two electrons from NADH to lipid-soluble ubiquinone and translocation of four protons (H^+^) from the matrix to the mitochondrial intermembrane space to generate a proton gradient that is the energy source in ATP synthesis. Metformin could decrease intracellular ATP production through the disruption of mitochondrial complex I (127), leading to a change in the ratio of AMP or ADP/ATP in an AMPK-independent pathway (81). Since gluconeogenesis is an energy-consuming process, it has been proposed that reduction of cellular ATP levels by metformin will lead to the suppression of hepatic gluconeogenesis (81). However, the ratio of AMP or ADP/ATP is affected only at >250 μM metformin (81, 119), which is unreachable in the portal vein at pharmacologic metformin concentrations (80 μM). Moreover, the cellular ADP/ATP ratio is not changed after metformin treatment in some studies (128). Recent study also supports the notion that metformin increases cellular AMP levels through the inhibition of AMP deaminase (129), which converts AMP to inosine monophosphate (IMP) and releases an ammonia molecule. The accumulated cellular AMP after metformin treatment results in the inhibition of adenylate cyclase, in turn reducing cAMP levels, leading to decreased PKA kinase activity and the inhibition of glucagon-dependent gluconeogenesis (130). More recently, metformin-induced increases in AMP concentrations have been shown to allosterically inhibit FBPase (124), which is a key rate-controlling enzyme in the gluconeogenic pathway by catalyzing the irreversible hydrolysis of F1,6BP to fructose-6-phosphate (Figure 1). Individuals with FBP1 deficiency present with hypoglycemia and metabolic acidosis due to impaired gluconeogenesis.

Recently, an additional mitochondrial target implicated in metformin has been proposed (131, 132). Metformin is reported to suppress mitochondrial glycerophosphate dehydrogenase (mGPDH), which is a key component of the α-glycerophosphate shuttle, resulting in an inhibition of NADH shuttle from the cytoplasm into mitochondria and the conversion of lactate to pyruvate. Subsequently, the hepatic gluconeogenesis from lactate is inhibited (Figure 5). This metformin pathway may be important in diabetes patients with high levels of serum lactate. However, as discussed in an editorial (133), the malate-aspartate shuttle is quantitatively much more important than the glycerophosphate shuttle in hepatocytes; thus, disruption of the glycerophosphate shuttle alone may not be enough to sustain impact on gluconeogenesis unless the mitochondrial membrane potential also becomes suppressed.

## Conclusion

Hepatic gluconeogenesis is largely controlled by transcriptional regulation of key rate-limiting enzymes, and these transcription regulators are mediated by hormonal signals in response to nutritional changes during the starvation-feeding cycle. Significant progress has been made to understand the expression regulation of gluconeogenic enzymes PEPCK and G6Pase by various transcriptional factors and their posttranslational modifications including phosphorylation, methylation and acetylation. Although PEPCK and G6Pase are regarded as the central operators of gluconeogenesis, recent investigations question their roles in the regulation of gluconeogenesis. Targeted deletion of the PEPCK gene in mice only led to a 40% reduction in gluconeogenic flux despite a 90% reduction in hepatic PEPCK expression (123). Moreover, although the expression of PEPCK and G6Pase are not increased in the poorly-controlled type 2 patients, their fasting blood glucose concentrations are dramatically elevated (122). These studies suggest that PEPCK and G6Pase may not be the most important regulators of gluconeogenesis. In addition to PEPCK and G6Pase, PC, and FBPase are also unique enzymes in gluconeogenesis; however, the regulation of their activities and expressions are rarely studied. Understanding the transcriptional regulation of PC and FBPase and their effects on gluconeogenesis should be the focus of future work.

Hepatic gluconeogenesis is a complex and genetically heterogeneous process modulated on multiple levels (1). Although there are growing data on the identification of transcription control of gluconeogenesis, which is a relatively slow process, limited information is available regarding the acute control of gluconeogenesis. Indeed, hepatic gluconeogenesis is suppressed rapidly following the intravenous administration of either galegine or metformin in rat; this is inconsistent with that of transcriptional regulation mechanisms. Regulation of hepatic gluconeogenesis can also be controlled through the regulation of substrate supply and allosteric regulation of enzyme activity. For example, glucagon can stimulate hepatic gluconeogenesis by inhibiting the liver-specific PK and induces the phosphorylation of the fructose-2,6-bisphosphatase 2 (PFK2). Phosphorylated PFK2 functions as phosphatase to decrease the production of fructose-2,6-bisphosphate, resulting in an inhibitory attenuation to the gluconeogenic enzyme FBPase. Additionally, phosphorylated PFK2 suppresses GK by promoting its nuclear translocation. Overall, identifying key regulators that control gluconeogenesis could provide novel ways to treat type 2 diabetes.

## Author Contributions

All authors listed have made a substantial, direct and intellectual contribution to the work, and approved it for publication.

### Conflict of Interest Statement

The authors declare that the research was conducted in the absence of any commercial or financial relationships that could be construed as a potential conflict of interest.
